# Assessing children's cognitive flexibility with the Shape Trail Test

**DOI:** 10.1371/journal.pone.0198254

**Published:** 2018-05-31

**Authors:** Amy Y. C. Chan, Sarah-Jane Morgan

**Affiliations:** School of Psychology, University of Wollongong, North Wollongong, New South Wales, Australia; University College London, UNITED KINGDOM

## Abstract

In this paper we report an initial validation of the Shape Trail Test–Child Version (STT-CV) with a non-clinical sample of children aged 6 to 9 years. The STT-CV has been developed as an age-appropriate and culturally fair direct downward extension of the Trail Making Test (TMT) for the assessment of cognitive flexibility. Children completed the STT-CV and four established measures of executive functions that assessed working memory, inhibitory control and task switching. Results showed the expected age-based differences in completion times for both parts of the STT-CV (Trail A and Trail B). Children’s performance on the STT-CV correlated significantly with all four measures of executive functions. After controlling for the effects of chronological age, completion times for Trail B remained correlated with most other measures of executive functions. These findings provide emerging evidence for the utility of the STT-CV, and highlight the need for designing and using appropriate variants of the TMT in the behavioural assessment of cognitive flexibility in developmentally and culturally diverse populations.

## Introduction

Executive function entails a broad class of mental processes that enable individuals to attend to relevant information and to respond appropriately to current task demands. It reflects the efficiency in three core areas of cognitive competence: working memory (temporarily holding and processing information in mind), inhibitory control (controlling one’s attention or behaviour so as to refrain from responding impulsively or producing a prepotent but inappropriate response), and cognitive flexibility (adjusting one’s behaviour flexibly according to new demands in a problem) [1]. Effective executive functioning is crucial to the development of important academic and social skills in childhood and beyond, with children’s executive functions being implicated in the development of skills such as mathematics ability (e.g., [2, 3]) and emotion regulation (e.g., [4]).

The effectiveness of these cognitive processes has been associated with the functioning of prefrontal areas of the brain (e.g., [5, 6]). Behavioural and neuroimaging research has led to the recognition that the prefrontal areas are at least partly functional by early childhood (e.g., [7, 8]). Numerous studies have sought to examine the normal development of executive function in childhood, with many assessment tasks being reported to chart age-related differences in performance within specific tasks (e.g., [9, 10]), as well as correlations in performance across different tasks that purport to assess the various components of executive function [11, 12, 13]. For instance, by 2 years of age, children begin to show the ability to inhibit a prepotent response in order to produce an alternative, task-appropriate response (e.g., [14]), with substantial improvement in such ability in the preschool period (e.g., [15, 16, 17, 18]). By 4 years of age, children show clear evidence of their ability to engage both the storage and processing aspects of working memory in cognitive activities [19, 20]; they also show an emergent ability to respond to tasks that demand cognitive flexibility [20, 21, 22]. Despite evidence that the key components of executive function are in place by early childhood, the development of the effectiveness of these cognitive processes continues into middle childhood and beyond [3, 9, 11, 20].

Notably, in reconciling between earlier findings that suggested a lack of prefrontal functions in preadolescents and more current findings that indicate at least partial prefrontal functioning in early childhood, researchers are increasingly acknowledging the importance of using developmentally appropriate behavioural measures to assess the developmental trajectory of prefrontal skills during childhood [22, 23, 24]. A wide range of developmentally sensitive and well established measures is available for assessing preschool children’s executive function (for an overview of representative tasks appropriate for 2- to 6-year-old children and the relative difficulties of those tasks, see [22]). Relatively few developmentally appropriate behavioural measures are available for assessing the continued improvement in different facets of executive function in primary school age children.

In this paper, we sought to contribute to the knowledge regarding normal executive function development in childhood by reporting findings from an initial test of the Shape Trail Test–Child Version (STT-CV)–for the assessment of cognitive flexibility of children in their early primary school years. To foreshadow, the STT-CV is a downward extension of the Trail Making Test–Child Version [25, 26] and reflects key features of a Shape Trail Test that has been used as a culturally fair assessment tool of cognitive flexibility in elderly adults [27]. We focus on the development and assessment of cognitive flexibility because this component of executive function is expected to be supported by the more basic cognitive processes of inhibitory control and working memory [1, 28]. Inasmuch as other behavioural measures have been devised to assess behaviours associated with cognitive flexibility development (for a review, see [1]), we reason that the STT-CV may be a culturally fair, developmentally appropriate, as well as time- and cost-effective tool that can provide additional information on the development of cognitive flexibility in childhood, to complement other existing measures of this construct.

### Trail Making Test and its variants

A common paradigm for assessing cognitive flexibility involves examining individuals’ ability in task or set switching [1]. Within this paradigm, individuals are typically required to initially learn to respond in a certain way with a set of stimuli, and subsequently switch to respond in an alternative way to the same (or similar) stimuli. Cognitive flexibility is assessed by identifying the change in accuracy and/or speed of responding when the correct way of responding has been modified (i.e., the switch cost). Examples of representative tasks include the Wisconsin Card Sorting Task [29] and other variants of the card sorting task [3, 30]. Other researchers have developed customised tasks in their assessment of cognitive flexibility (e.g., [9, 20, 21]).

Consistent with the approach to examine cognitive flexibility via task switching performance, the Trail Making Test (TMT) is a widely used neuropsychological measure of mental flexibility and executive function [26]. This paper-and-pencil task assesses the individual’s ability to simultaneously keep in mind alphabetical and numerical sequences, and it only requires a few minutes to administer. The original Adult Version of the TMT entails drawing pencil lines quickly and accurately to sequentially connect a set of 25 encircled numbers that are randomly arranged on a page. In the first task, the circles have the numbers 1 to 25 embedded in them (Trail A). In a second task (Trail B), the circles contain the numbers 1 to 13 and the letters A to L, and the task requires connecting the numbers and letters in alternating order (1-A-2-B, … etc.). The Children’s Version of the TMT has been designed for children 9 to 14 years of age, and it contains 15 circles in both parts to reduce the complexity of the visual search required (i.e., the numbers 1 to 15 for Trail A, and the numbers 1 to 8 and the letters A to H for Trail B) [31].

A key merit of the TMT is the simplicity of its task instructions and time efficiency of administration, thus allowing it to be incorporated into a larger battery of measures to assess executive function more comprehensively. As an example of its utility, Trail B has been shown to correlate significantly with other measures of cognitive flexibility that may require longer task administration time (e.g., percentage of perseverative errors in the Wisconsin Card Sorting Task–[32]). Furthermore, reliable difference in TMT performance has been shown between individuals with brain damage and normal controls, both in adults [33] and children [25].

Despite its simplicity and utility, successful and efficient completion of the TMT requires the abilities to: (i) automatically recognise the symbolic significance of numbers and letters, (ii) visually scan the page continuously to identify relevant target stimuli, (iii) integrate the numerical and alphabetical series flexibly, and (iv) complete task requirements under the pressure of time [31]. At least by the middle primary school years, children in an English-speaking environment can be expected to have fully mastered the symbolic significance of the English alphabet and its applications so that they can recall it effortlessly while completing tasks such as the TMT. However, for younger primary school children and individuals with limited English language proficiency, their performance in Trail B would presumably reflect a combination of their efficiency in mental flexibility as well as their development in other aforementioned areas crucial for successful TMT task completion. Indeed, some studies have involved administering the Child Version of the TMT to 6-year-old children [34] or the Adult Version of the TMT to children [35], and have yielded rather slow task completion times, particularly for Trail B. Such results highlight the need for an appropriate further downward extension of the TMT and to validate it with children in their early to middle primary school years.

The Color Trails Test [36, 37] and Shape Trails Test (STT; [27]) have been designed to be culturally unbiased analogues of the traditional TMT. Part 1 of both of these tests are similar to Trail A in the traditional TMT. Part 2 of the Color Trails Test requires the test taker to connect numbers embedded in circles in two alternating colours (pink and yellow–e.g., Pink 1 –Yellow 2 –Pink 3 –Yellow 4, … etc.); and Part 2 of the STT requires connecting numbers embedded in two alternating shapes (circles and squares–e.g., Circle 1 –Square 2 –Circle 3 –Square 4, … etc.). Zhao et al. [27] propose that the STT is in line with the Color Trails Test as a time-efficient assessment tool for task switching competence; but given that the STT instead requires test takers to join two sets of numbered dots that are enclosed by squares and circles, the STT has the added benefit that it does not require test takers to have good colour vision, nor does it require test administrators to have access to colour printing to produce the stimuli. Performance in the Color Trails and STT has been shown to be significantly correlated to each other [27].

Zhao et al. [27] provided an initial validation of the STT with a large sample of elderly Chinese adults, and demonstrated that completion times for Parts A and B of the STT reliably differed across cognitively normal controls, patients with amnesic mild cognitive impairment and patients with Alzheimer’s Disease. Furthermore, these authors provided some evidence that the STT might be harder than the TMT or the Color Trail Test, and highlighted the need for further validation studies on the STT.

A notable departure of Part 2 of the Color Trails and STT from the traditional TMT is that in these tests, the display for Part 2 contains duplicates of every number in the first part, with each number embedded with both yellow and pink circles (Color Trails) and a square and a circle (STT). Thus in Part 2 of these tests, the visual scanning requirement is substantially increased because the test taker has to scan for correct responses efficiently from a visually more complex display that contains twice as many stimuli as that in Part 1. Hence, the greater time taken to complete Part 2 may not only represent switch cost, but also extra time taken to scan for appropriate stimuli in the more complex display.

### The present study

The aim of this study was to explore the suitability of the Shape Trail Test as a culturally fair and low-cost tool for assessing primary school age children’s task switching competence. It is noteworthy that age- and education-based norms for the TMT in adults aged 18–89 years have been developed as an index of normal cognitive flexibility development in adulthood [38]. To this end, if an age-appropriate and direct downward extension of the TMT for the early school years can be developed, it would enable the use of the same task paradigm across wider age ranges to gather age-related norms on the normal development of cognitive flexibility in early childhood and beyond.

To the extent that the STT is a sensitive tool for assessing individual differences in task switching, we reasoned that it should yield reliable age-related variations in the performance of normally developing children. We further reasoned that for the child version of the STT to be an appropriate analogue of the child version of the TMT, it would be important for it to eliminate the requirement for a child to readily recognise the symbolic significance of English letters, while preserving all other characteristics of the child version of the TMT (e.g., complexity of visual display and length of task).

We report findings from an initial test of the STT-CV in a non-clinical sample of 6- to 9-year-old children. Our focus was on the early school years because in a review of the normal development of the prefrontal cortex from infancy to young adulthood, the period from approximately 6–7 years to 11 years has been identified as one with marked changes in the abilities associated with the prefrontal cortex [5]. Specifically, the abilities to exercise inhibition and to hold information in mind and manipulating, monitoring, or transforming it are among those tied to changes to the density of neurons in the dorsolateral prefrontal cortex during this period [5]. The child version of the TMT is already available for the assessment of children 9 years of age and older. Hence, the STT-CV may have its greatest utility in characterising task switching performance in children aged 9 years and younger.

Consistent with proposals regarding the roles of more basic cognitive mechanisms in supporting the development of cognitive flexibility (e.g., [1, 28]), we evaluated children’s performance on the STT-CV against that on established measures of working memory, inhibitory control and task switching. Within the present study, we operationalised working memory capacity as task performance in the backward digit span task [39] and the counting span task [40]. Efficiencies of inhibitory control and task switching were operationalised as children’s performance in a Stroop-like task [15] and a card sort task [15], respectively.

Two complementary issues have had methodological implications on our assessments of inhibitory control and task switching. First, Macdonald et al. [24] reported an age-related increase from 5 to 8 years in children’s self-corrections of errors in inhibitory control tasks. These authors highlighted that part of what develops in inhibitory control during this period is a greater consciousness of errors. Second, Davidson et al. [20] have noted that when young children and adults are subjected to the same executive function tasks, effects seen in differences in response times in adults are typically manifested as the same effects seen in differences in accuracy in young children. These authors therefore highlighted the importance of examining both speed and accuracy of children’s executive function task performance where possible. Consequently, within the inhibitory control and card sort tasks in the present study, we used a composite performance index (“scaled completion time”–see Method for further details) that took into account different “grades” of accuracy in children’s responses, as well as their time efficiency of task completion.

We hypothesised that:

H1a: Children would show a switch cost when the task requires alternating between numbered dots enclosed by squares and circles (Trail B) as opposed to joining numbered dots enclosed by circles only (Trail A) (i.e., completion time for Trail B would be longer than that for Trail A); and

H1b: The switch cost in completing the STT-CV would reduce with age.

H2: Children’s performance in the STT-CV would correlate significantly with their performance in other executive function measures (working memory, inhibitory control and another task switching measure).

## Method

### Participants

This study was part of a larger research project on cognitive and emotional development. The sample comprised 68 children who were recruited either from one school in central western New South Wales (*n* = 22) or through social media (*n* = 46). There were 17 children aged 6 years (*M* = 75.94 months, *SD* = 3.47, 6 girls, 11 boys); 17 children aged 7 years (*M* = 89.53 months, *SD* = 2.92, 10 girls, 7 boys); 19 children aged 8 years (*M* = 101.53 months, *SD* = 2.59, 14 girls, 5 boys); and 15 children aged 9 years (*M* = 112.67 months, *SD* = 3.52, 8 girls, 7 boys). Potential participants’ parents were contacted via the school and social media platforms to invite children aged 6 to 9 years for their voluntary participation. All children who met the age criterion and returned a signed parental consent form were included in the study. The study was further explained verbally in age-appropriate terms to each child at the outset of each session, and verbal consent was gained from each child before participation in each session. All children spoke fluent English and only one child spoke a second language (Japanese) in addition to English. This study was approved by the University of Wollongong Human Research Ethics Committee (research protocol HE16/065).

### Materials and procedure

This study comprised five tasks, including a measure of inhibitory control (Day/Night task), two measures of working memory (Backward Digit Span Task and Counting Span Test), and two measures of attentional switching (Card Sort task and Shape Trail Test). Every participant completed all tasks. The tasks were administered in a fixed order for all participants over two individual sessions separated by three to seven days apart. Participants completed the Day/Night task followed by the Card Sort task and Counting Span task in Session 1, and the Backward Digit Span task followed by the STT-CV in Session 2.

The same female experimenter conducted all testing sessions. At the outset of each session, a brief oral introduction was given to the participant and verbal consent was sought from each participant. Demographic information, such as date of birth, gender, age, and year at school was obtained from the parents/carers for each participant prior to the sessions.

#### Day/Night task [15]

The experimenter verified that the participant associated the sun with daytime and the moon with night-time. The participant was then shown a black card with a white moon and stars on it, and was instructed that each time they saw this card they were to say ‘day’. They were then shown a white card with a yellow sun on it and were instructed that each time they saw this card they were to say ‘night’. Two practice trials followed in which the child was shown one of each card. All children answered correctly without the need for the experimenter to repeat the rules for each card and the practice trials. Following this, 16 test trials were run in a fixed random order with no feedback on errors, and the total time taken (to the nearest hundredth of a second) to complete all 16 trials was recorded. To calculate accuracy scores for the Day/Night Task, participants received a score of 0 for an incorrect response, 1 for a response that was initially incorrect but subsequently self-corrected, and 2 for a correct response. Scores for the trials were then summed to create a composite score with a maximum possible total of 32. We calculated an overall index of task performance by dividing the total completion time by the composite accuracy score on this task (scaled completion time). Thus a lower score would indicate better performance.

#### Counting span test [40]

This test comprised five levels with each level containing three sets of items. The items for this test were white cards on which were printed red dots (the target items) along with yellow, green and blue distractor dots. There were 18 dots randomly placed on each card, with between 2 to 6 of these dots red. The card sets varied in size from one card per set (Level 1) to five cards per set (Level 5). The participant was required to count the red dots only on each card by pointing to the dots and counting aloud. After the participant counted the dots, each card was turned face down and the next card in the set was presented for counting. After the last card in the set was turned face down, the participant was asked to recall the number of dots counted on each card in order from first card to last. To proceed to the next level, the participant had to have correctly recalled at least two of the three sets comprising the preceding level. Responses were scored as 0 for an incorrect response, and 1 for a correct response on each trial. The scores for each trial were then summed to create a composite accuracy score with a maximum possible total of 15.

#### Digit Span Backward [39]

The Digit Span Backward subtest of the Australian version of the Wechsler Intelligence Scale for Children, Fourth Edition (WISC-IV; [39]) was used. In this task, the participant listened to the experimenter reading a sequence of digits out loud (e.g. *6*, *8*) and then was to repeat the sequence of numbers backwards out loud (the correct response would be *8*, *6*) to the experimenter. This task comprised 8 levels with two trials per level. Levels 1 and 2 consisted of two numbers per trial; level three trials comprised three numbers per trial; finishing with eight-number sets in the level eight trials. The task was discontinued if the participant was incorrect on both trials on a level. Responses were scored as 0 for an incorrect response, and 1 for a correct response on each trial, with scores for each trial then summed to create a composite accuracy score with a maximum possible total of 16.

#### Card sort task [15]

The participant was shown two same-sized silver boxes with slots cut into the lids. One box had a picture of a red rabbit attached and the other had a picture of a blue boat attached. The experimenter then produced a stack of five cards depicting red rabbits, blue rabbits, red boats, and blue boats. The participant was instructed to sort all of the cards according to their shape, with rabbits going into the rabbit container and boats going into the boat container. After demonstrating with one of each card, the experimenter asked the participant to sort five cards in a fixed set order. Errors were not corrected, but were recorded and scored as per the Day/Night task. The total time taken (to the nearest hundredth of a second) to sort the cards was also recorded. Afterwards, the experimenter asked the participant to sort the cards by colour: all the blue cards into the blue box and all of the red cards into the red box. Five post switch trials then followed with the experimenter recording errors and the total time taken to sort the cards in the same manner as for the pre-switch trials. Scores for the responses were summed for each of the pre- and post-switch trials to form a composite accuracy score for pre-switch trials and for post switch trials, each with a maximum possible score of 10 (indicating 5 correct answers). For this task, we focussed on children’s performance on the post-switch trials. As per the Day/Night task, we calculated an overall performance index of the Card Sort task (scaled completion time) by dividing participants’ completion time by their accuracy score on post-switch trials.

#### Shape Trail Test–child version (STT-CV; adapted from [27])

The STT-CV has been adapted specifically for this study to produce a child version by mirroring the number and position of items in the child version of the TMT [31]. This test requires basic numerical knowledge but does not require any knowledge of the English alphabet. The STT-CV comprises two trails printed on A4-size sheets; Trail A and Trail B. Trail A is identical to Part A of the Child Version of the TMT, which displays a sequence of numbers from 1 to 15, with each number enclosed by a small circle. Trail B (S1 Fig) displays the numbers 1 through 8 enclosed by small circles and the numbers 1 through 7 enclosed by small squares.

Instructions were given to the participant as per the instruction manual for the TMT–Child Version [31] and the participant completed a sample page for Trail A containing eight items, the experimenter stopping the participant for corrections if they made any mistakes. Participants were then asked to complete Trail A as quickly as possible while trying not to make any mistakes. The time taken to complete the trail (to the nearest hundredth of a second) was recorded.

Afterwards, the experimenter showed a sample page for Trail B containing eight items to the participant to complete, informing the participant that that the rules have now changed and they were now to draw a line alternating between the circles and squares in sequential numerical order (1 in the circle, 1 in the square, 2 in the circle, 2 in the square, … etc.). The experimenter stopped the participant for corrections if they made any mistakes. Participants then completed Trail B, working as quickly as possible while trying not to make any mistakes. The time taken to complete the trail (to the nearest hundredth of a second) was recorded.

## Results

**Fig 1:**
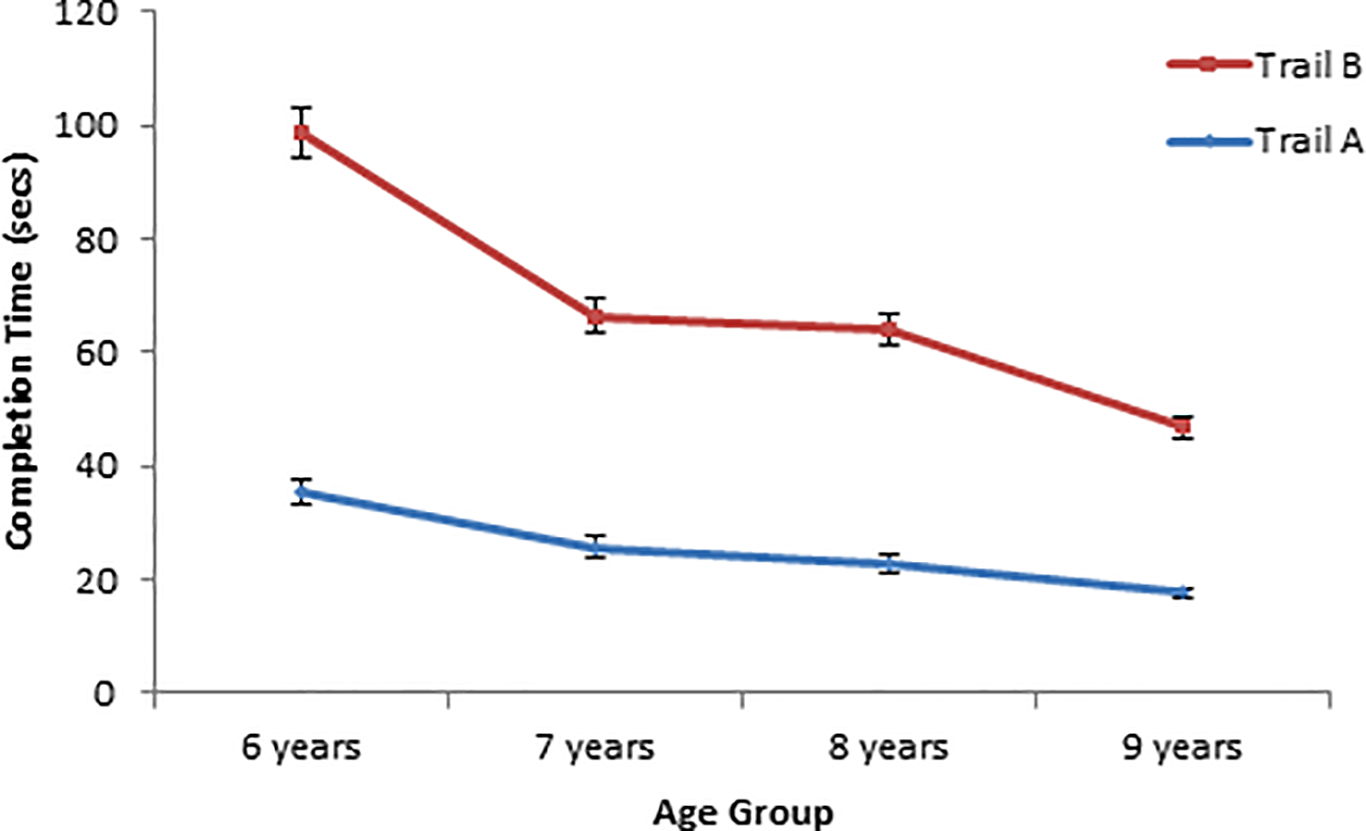
Mean completion times for Trail A and Trail B in the Shape Trail Test–child version for each age group. Error bars indicate one standard error of the mean.

The pattern of children’s performance in Trail A and Trail B of the STT-CV is shown in Fig 1. Table 1 shows the descriptive statistics on children’s performance on all other measures in the present study. As expected, older children performed better than younger children on all tasks. There was a statistically significant effect of age group on the key performance index for each of the Day/Night task [*F*(3, 64) = 10.72, *p* < .001, η^2^ = .33], Backward Digit Span task [*F*(3, 64) = 4.79, *p* = .004, η^2^ = .18], Counting Span task [*F*(3, 64) = 7.05, *p* < .001, η^2^ = .25], and Card Sort task [*F*(3, 64) = 3.43, *p* = .02, η^2^ = .14]. Notably, as per the scaled completion time data for the Card Sort task, younger children were less efficient in accomplishing the same ceiling-level accuracy compared to their older counterparts. By comparison, for the Day/Night task, while 6-year-old children were slower in their responses and showed relatively high proportions of incorrect and self-corrected responses, children at the older age levels showed predominantly correct and self-corrected responses and completed the task more efficiently (see Table 1).

**Table 1:** Summary of means [and standard deviations] for the major study variables.

	Age group			
	6 Years (n = 17)	7 Years (n = 17)	8 Years (n = 19)	9 Years (n = 15)
**Day/Night task**				
Completion time (secs)	32.34 (3.70)	26.89 (5.09)	26.13 (4.01)	22.98 (2.58)
Accuracy	26.35 (4.87)	29.59 (1.97)	29.47 (3.01)	29.73 (2.69)
Proportion of responses				
Incorrect	.11 (.12)	.03 (.04)	.04 (.08)	.02 (.05)
Self-corrected	.13 (.12)	.08 (.07)	.09 (.08)	.11 (.10)
Correct	.76 (.20)	.88 (.09)	.88 (.12)	.88 (.13)
Scaled completion time	1.29 (.42)	.91 (.18)	.91 (.24)	.79 (.16)
**BDS accuracy**	4.53 (1.33)	5.65 (1.54)	6.58 (2.29)	6.33 (1.54)
**Counting span accuracy**	6.82 (1.38)	8.47 (2.03)	9.21 (2.15)	9.67 (1.99)
**Card sort task**				
Pre-switch trials				
Completion time (secs)	8.24 (3.34)	6.71 (1.91)	6.48 (1.98)	5.50 (1.42)
Accuracy	9.88 (.33)	9.71 (.59)	9.89 (.32)	9.80 (.56)
Post-switch trials				
Completion time (secs)	7.83 (3.27)	6.39 (2.15)	5.55 (1.52)	5.04 (1.77)
Accuracy	9.71 (.99)	9.94 (.24)	9.63 (1.38)	9.87 (.35)
Proportion of responses				
Incorrect	.01 (.05)	.00 (.00)	.03 (.14)	.00 (.00)
Self-corrected	.04 (.11)	.01 (.05)	.01 (.05)	.03 (.07)
Correct	.95 (.15)	.99 (.05)	.96 (.14)	.97 (.07)
Scaled completion time	.84 (.45)	.65 (.24)	.60 (.25)	.51 (.18)

BDS = Backward Digit Span task; Scaled completion time = Completion time divided by accuracy score

In the STT-CV, both Trail A and Trail B showed the predicted decrease in completion times with age. To test for an age-related reduction in the switch cost in completing the two parts of the STT-CV, we conducted a 4 (age group) x 2 (STT trial) mixed design analysis of variance (ANOVA) on participants’ completion times for Trail A and Trail B. As predicted, the main effects of age group [*F*(3,64) = 23.56, *p* < .001, η^2^_p_ = .53] and STT trial [*F*(1,64) = 162.64, *p* < .001, η^2^_p_ = .72] were both statistically significant. Averaged across age groups, children were faster in completing Trail A (*M* = 25.55 secs, *SD* = 9.52) than Trail B (*M* = 44.00 secs, *SD* = 17.96). These main effects were qualified by a significant age group x STT trial interaction, [*F*(3,64) = 6.05, *p* = .001, η^2^_p_ = .22]. Pairwise comparisons between Trail A time and Trail B time within each age group (with Bonferroni-adjusted α at .05) indicated that for all age groups, children took significantly longer to complete Trail B than they did for Trail A (all *p*s < .001). More importantly, as age level increased, there was a general reduction in the mean difference between the completion times for Trail A and Trail B (see means in Fig 1). These findings were in support of H1.

Next, we examined the correlations among age and children’s performance on the STT-CV and other executive function measures. To account for the relatively small sample size of the present study, we used a bootstrapping procedure [41] with 1000 iterations to generate the bias corrected and accelerated (BCa) 95% confidence interval for each correlation.

The results are shown in Table 2. Children’s performance on all of our executive functioning measures was significantly correlated with one another in the expected direction. Not surprisingly, age was significantly correlated with performance on all measures in our study. Comparing our two task switching measures (i.e., Card Sort and the STT-CV), the STT-CV variables correlated more strongly with performance on the Day/Night, Backward Digit Span and Counting Span tasks.

**Table 2:** Summary of means, standard deviations, and intercorrelations [and 95% confidence intervals] for age and performance indices on inhibitory control, working memory and cognitive flexibility measures.

	M	SD	2	3	4	5	6	7
1. Age (in months)	94.59	13.82	-.52** [-.63, -.45]	.37** [.19, .55]	.49** [.29, .65]	-.40** [-.54, -.25]	-.69** [-.78, -.58]	-.59** [-.71, -.46]
2. Day/Night	0.98	0.33	-	-.37** [-.48, -.25]	-.39** [-.54, -.22]	.39** [.13, .63]	.46** [.30, .65]	.53** [.30, .70]
3. BDS accuracy	5.78	1.88		-	.48** [.23, .66]	-.30* [-.51, -.04]	-.41** [-.56, -.23]	-.47** [-.61, -.30]
4. Counting span accuracy	8.53	2.16			-	-.27 * [-.42, -.11]	-.55** [-.69, -.40]	-.43** [-.48, -.27]
5. Card Sort	0.65	0.32				-	.47** [.14, .70]	.42** [.12, .65]
6. STT-CV Trail A	25.55	9.52					-	.71** [.56, .84]
7. STT-CV Trail B	44.00	17.96						-

BDS = Backward Digit Span task; STT-CV = Shape Trail Test—Child Version

** p < .01

* p < .05

We conducted a further correlation analysis on the relationships among the executive functioning variables after controlling for variability associated with children’s chronological age. The partial correlations (see Table 3) indicated that Trail B completion time remained significantly correlated with performance in the Day/Night and Backward Digit Span tasks, and its correlation with performance in the Counting Span task was marginally significant. However, Trail A completion time and Card Sort task performance were no longer correlated with the other executive function measures. On the basis of the bootstrapped confidence intervals (refer to Table 3), the correlations of Card Sort task performance with Trail A and Trail B completion times were not statistically reliable either. These findings thus provided partial support for H2.

**Table 3:** Summary of partial intercorrelations [and 95% confidence intervals] for performance indices on inhibitory control, working memory and cognitive flexibility measures.

	2	3	4	5	6
1. Day/Night	-.22# [-.38, -.05]	-.18 [-.35, .04]	.23# [-.01, .48]	.18 [-.01, .35]	.33** [.03, .53]
2. BDS accuracy	-	.36** [.07, .57]	-.18 [-.40, .10]	-.22# [-.42, -.01]	-.33** [-.50, -.15]
3. Counting span accuracy		-	-.09 [-.26, .07]	-.35** [-.57, -.06]	-.21# [-.38, -.03]
4. Card Sort			-	.29* [-.06, .60]	.25* [-.11, .52]
5. STT-CV Trail A				-	.52** [.29, .72]
6. STT-CV Trail B					-

BDS = Backward Digit Span task; STT-CV = Shape Trail Test—Child Version

** p < .01

* p < .05

# p < .10

## Discussion

The Shape Trail Test has been identified as a time-efficient and culturally fair analogue of the traditional TMT in the assessment of task switching competence, a core component of executive function [18]. In the present study, we developed and tested a child version of the STT that included key features of the adult version of the STT while also preserving important features of the child version of the traditional TMT. Consistent with previous research on the STT [27], the development of the STT-CV has been guided by the assumption that by 6 years of age, children are competent in discriminating between basic geometric shapes (square vs. circle) and that they have developed basic numerical literary for counting. Normally developing 6- to 9-year-old children showed reliable age-based differences in performance on both Trail A and Trail B of the STT-CV. As expected, the switch cost in completion time from Trail A and Trail B decreased as age increased. Our data suggest that the STT-CV may be a valid assessment task to observe the development of task switching competence in the early school years. Furthermore, children’s performance in the STT-CV correlated significantly with measures of working memory, inhibitory control and task switching, thus providing early evidence of the construct validity of the STT-CV.

However, we also found that once children’s chronological age was statistically controlled for, some of the observed correlations were no longer statistically reliable. Although this finding may simply highlight the influence of general age-related gains in executive function competence on children’s performance across various relevant measures, the diminished correlation between the STT-CV and the card sort task was somewhat unexpected, because both tasks were supposed to measure children’s cognitive flexibility via their task switching performance. When age-based variations in task performance was statistically controlled for and a more stringent criterion for assessing the reliability of correlations was used (i.e., using bootstrapped confidence intervals), the positive relationships of children’s STT-CV performance in Trail A and Trail B with their performance on the Card Sort task was no longer clearly evident. A potential explanation is that the small number of items included in the card sort task might have restricted the range of observable individual differences in this task. Further validation studies are needed to determine the relationship of the STT-CV with other developmentally appropriate measures of children’s cognitive flexibility. This could potentially involve a modified version of the card sort task that includes additional pre-switch and post-switch items, to enable more scope for individual differences in responding to be adequately captured (e.g., [3]). It would be beneficial to assess children’s performance on the STT-CV against other established indices of cognitive flexibility also (e.g., the number of perseverative errors on the Wisconsin Card Sorting Task).

A limitation of the present study was that we did not concurrently assess children’s performance on the STT-CV against that on other trails tests such as the Color Trails or the child version of the traditional TMT. However, it is noteworthy that children across all age levels within our sample could fully understand the task instructions and successfully completed both parts of the STT-CV, with both parts of this task showing expected individual differences within and across the four age levels. Moreover, Trail A and Trail B completion times for our subsample of 9-year-old children (see full data set in S1 File) were consistent with the distributions of completion times for normally developing 9- to 14-year-old children on the child version of the TMT (see [25]). The requirement for further validation of the STT-CV notwithstanding, our data provide initial evidence that the STT-CV may be a useful direct downward extension of the child version of the TMT. Further research is warranted to examine the psychometric properties of the STT-CV more closely. Another limitation was that the present study involved a relatively small convenience sample of Australian children. We did not gather information on each child’s ethnicity or the family’s socioeconomic status. Although we did not expect these factors to influence children’s performance on the STT-CV, further research is needed to establish the population-based and cross-cultural generalisability of the current findings.

An implication of the current findings is that the STT-CV may provide a sensitive measure of children’s developing cognitive flexibility in a way that is intended in the traditional TMT. This is because this variant of the traditional TMT does not have extra visual search demands or assume immediate recognition of the symbolic significance of the English alphabet. Therefore the switch cost from Trail A to Trail B cannot be attributed to difficulties with cognitive demands beyond those required for monitoring and shifting.

A further implication of our findings is that the STT-CV has the potential to be included as part of a larger test battery for the behavioural assessment of executive function development. There is substantial theoretical interest in the operationalisation of the cognitive flexibility construct in behavioural paradigms (e.g., [28]). Furthermore, there is practical interest in the roles of components of executive function in other important areas of development such as mathematics skills [2, 3] and major health and wealth outcomes later in life [42]. All of these avenues of enquiry would typically entail children being assessed on multiple tasks that index the relationship between relevant executive function components and criterion variables. All else being equal, it would be desirable to include briefer tasks that can help to keep the length of the testing procedure as short as possible. In this paper, we have provided emerging evidence that the STT-CV is an effective assessment tool to complement the traditional TMT to chart the development of cognitive flexibility from the early school years onwards. Further research is needed to replicate the present findings with a larger sample, and to develop appropriate age norms for the STT-CV. Furthermore, given that the STT-CV has a simple task format that children can readily understand, it would be useful to explore the appropriateness of the STT-CV for assessing task switching performance in children younger than 6 years of age also.

In summary, our results suggest that the STT-CV is a meaningful assessment tool of the development of cognitive flexibility in the early school years. This task may complement the traditional TMT and other available measures to contribute to knowledge on children’s growing ability to monitor and shift their responses flexibly as per current task demands. Closer examination in further research is required to establish the utility of this task in assessing the development of cognitive flexibility in normally developing children across different cultural and education backgrounds, as well as in clinical populations.

## Supporting information

S1 Fig Trail B of the Shape Trail Test–child version. (PDF)

S1 File Raw data file for the present study. (XLSX)
