# Supplementary material for: Assessing children's cognitive flexibility with the Shape Trail Test
Source: PLoS One. 2018 May 31;13(5):e0198254. doi: 10.1371/journal.pone.0198254 (PMC5979013; doi:10.1371/journal.pone.0198254)
Supplement: S1 Fig — (PDF) [file pone.0198254.s001.pdf]

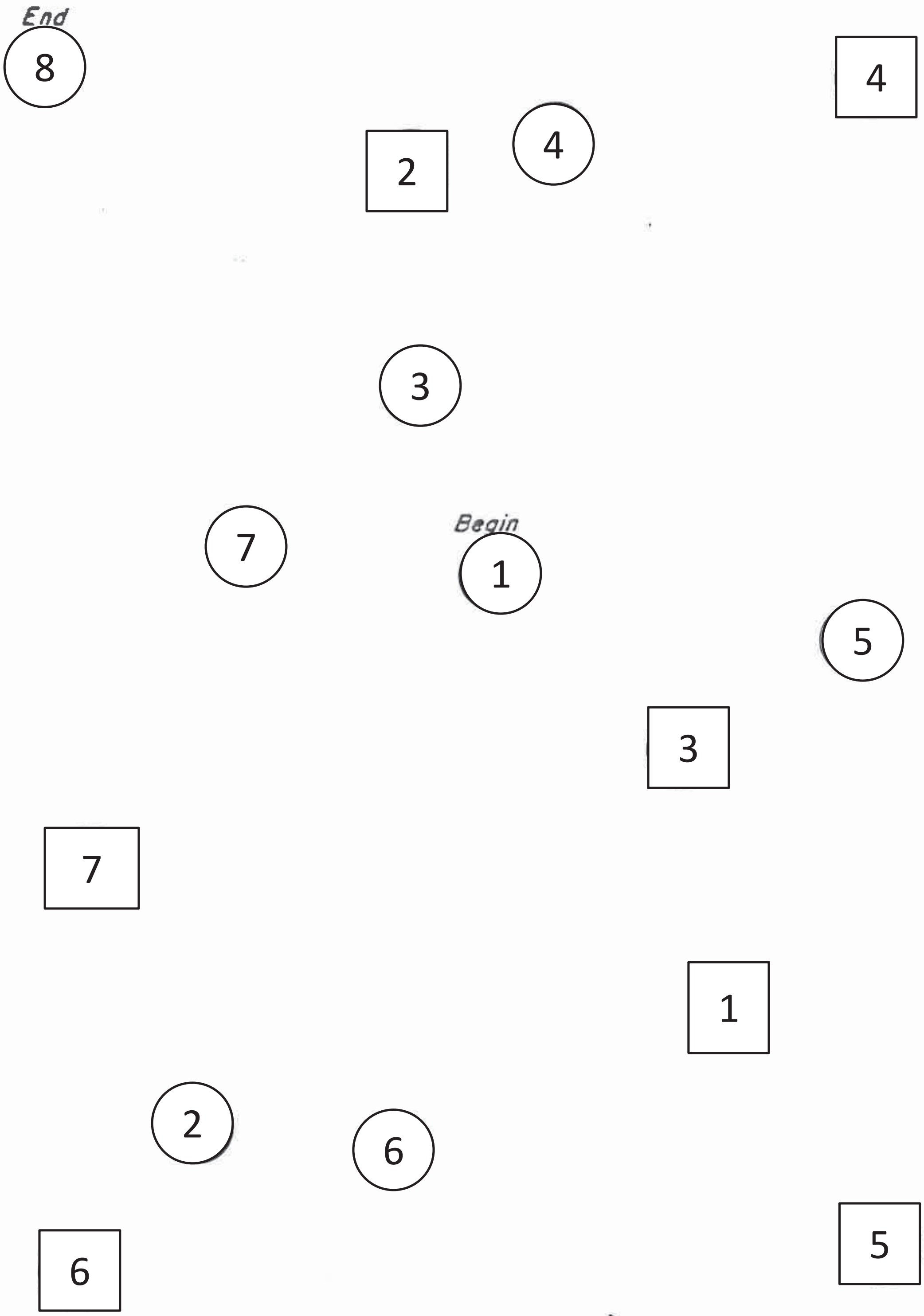
End
8
4
2
4
3
Begin
7
1
5
3
7
1
2
6
5
6
